# Aluminum-free glass ionomer cements containing 45S5 Bioglass^®^ and its bioglass-ceramic

**DOI:** 10.1007/s10856-021-06553-3

**Published:** 2021-06-22

**Authors:** Alireza Zandi Karimi, Ehsan Rezabeigi, Robin A. L. Drew

**Affiliations:** 1 Department of Mechanical, Industrial and Aerospace Engineering, Concordia University, Montréal, QC H3G 1M8 Canada; 2 Department of Mining and Materials Engineering, McGill University, Wong Building, 3610 Rue University, Montréal, QC H3A 0C5 Canada

## Abstract

Although the incorporation of bioactive glasses into glass ionomer cements (GICs) has led to promising results, using a bioactive glass as the only solid component of GICs has never been investigated. In this study, we developed an Al-free GIC with standard compressive strength using various combinations of 45S5 Bioglass^®^ and its glass-ceramic as the solid component. The glass-ceramic particles with 74% crystallinity were used for this purpose as they can best act as both remineralizing and reinforcing agents. Strengthening mechanisms including crack deflection and crack-tip shielding were activated for the GICs containing 50–50 wt% bioglass and bioglass-ceramic as the optimum ratio. The progression of the GIC setting reaction at its early stages was also monitored and verified. We also discussed that our bimodal particle size distribution containing both micron- and nanosized particles may enhance the packing density and integrity of the structure of the cements after setting. In such GICs produced in this study, the toxic effects of Al are avoided while chemical bonds are expected to form between the cement and the surrounding hard tissue(s) through interfacial biomineralization and adhesion.

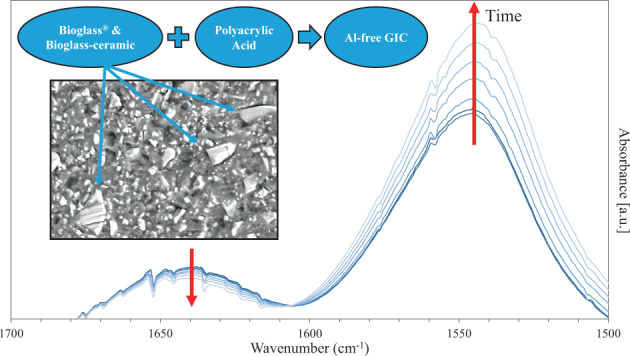

## Introduction

Glass ionomer cements (GICs), also known as glass polyalkenoates, were developed and patented in 1969 at the Laboratory of the Government Chemist (London, UK) [1]. Since then, GICs have been extensively studied and used as luting, sealing and restorative materials in modern dentistry [2, 3]. GICs exhibit many clinical advantages compared to other restoratives including the ability to develop a physicochemical bond to hard tissues, low coefficient of thermal expansion, desirable translucency and proper biocompatibility with the pulp tissue and primary cultures of bone cells—all of which can significantly promote their in vivo performance [4–6].

GICs are classified as acid-base cements consisting of an aqueous solution of a polyalkenoic acid and a powdered calcium aluminosilicate glass component with a basic nature [7]. The polyalkenoic acid is a family of complex acids normally including polyacrylic acid (PAA) or an acrylic/maleic/itaconic acid copolymer. During the acid-base setting reaction, multivalent counterions (e.g., Ca^2+^ and Al^3+^) leach out from glass particles resulting in the crosslinking of polyalkenoic acid chains [4, 8], which leads to cement hardening.

In addition to the field of dentistry, GICs have been considered as a potential substitute for polymethylmethacrylate (PMMA) bone cements for orthopedic applications due to their ability to chemically bond to hydroxyapatite (HA) [9]. Unlike PMMA-based bone cements, GICs exhibit good adhesion to hard tissue, proper stability in an aqueous environment, low shrinkage and low exothermic setting reaction [10, 11]. Despite all the advantages, the reliance of GICs on the presence of Al has restricted their application in orthopedics and dentistry. Al ion is recognized as a neurotoxin which promotes cellular oxidation [12] and disrupts cellular homeostasis [13]. There have been reports of aluminum traces in the brain, cerebral spinal fluid, blood and urine up to 77 days after implanting an aluminum-containing bone cement [14]. This issue has also been implicated in and linked to the pathogenesis of many neurological disorders such as Parkinson’s and Alzheimer’s diseases [15]. Due to the concerns about the release of Al ions from GICs, there has been a growing interest towards the development of Al-free glass ionomer cements for both dentistry and orthopedics applications [14]. Also, the existing formulations of conventional GIC suffer from poor mechanical properties limiting their applications in high stress-bearing areas of the body. Attempts have been made to overcome this drawback including the incorporation of glass fibers [16] or metallic oxide (nano-) particles [17]; glass composition modification [13] and using novel polyacids [18]. In our previous work [19], it was shown that the incorporation of an optimum amount of 45S5 bioglass-ceramic with a certain degree of crystallinity containing mechanically strong combeite (Na_2_Ca_2_Si_3_O_9_) phase can improve the mechanical performance of GICs while enhancing their remineralizing properties [19].

GICs can be considered bioactive since they can release ions via dissolution of calcium aluminosilicate glass particles causing certain biomineralization within their clinical environment [20]. Improving GICs remineralizing properties can potentially lead to a stronger chemical bond between the native tissue(s) and the cement in vivo. Although the incorporation of various bioactive glass or ceramic fillers into GICs has been investigated for this purpose [19, 21–23], utilizing bioactive glass particles as the whole solid powder component of the cement has never been previously studied. If the used bioactive glass in such a GIC transforms into HA in a controlled manner, the hard tissues surrounding the GIC can take advantage of the resulting interfacial biomineralization and adhesion [20].

45S5 Bioglass^®^ (45 wt% SiO_2_, 24.5 wt% Na_2_O, 24.5 wt% CaO, 6 wt% P_2_O_5_) is known to be the first commercial bioactive glass [24]. Developed by Hench in the late 70’s, this bioglass was the first material that was able to form a stable bond with tissues through the formation of an apatite layer [24, 25]. 45S5 Bioglass^®^ has similar characteristics to the common GIC glass component. For instance, it contains a high volume of network modifiers, CaO and Na_2_O, which can break the continuous Si–O–Si structural units resulting in the formation of non-bridging oxygens [26]. Upon incubation in an aqueous environment, 45S5 Bioglass® participates in the cation exchange of Na^+^, Ca^2+^ with H^+^ from the solution [21]. When this bioglass is mixed with polyacid solution, the released Ca^2+^ ions may be able to react with the polyacrylate chains creating an ionically crosslinked polyacrylate matrix [26, 27], similar to the setting reaction in conventional GICs. Furthermore, PO_4_^3-^ is released from the Bioglass^®^ as a result of reacting with the polyacid [21]. It has been shown that the existence of phosphorous (along with Si) within the matrix results in formation of an inorganic network which interpenetrates with the aforementioned Ca^2+^ polyacrylate matrix [28]. Formation of this network contributes to the insolubility of GICs as well as their gradual increase in compressive strength with time [27].

Due to its high bioactivity index [25], 45S5 Bioglass^®^ can be overreactive to the polyacid resulting in a premature setting. Alternatively, 45S5 glass-ceramic with appropriately lower bioactivity (i.e., reaction/dissolution rate) and superior mechanical properties compared to those of the 45S5 Bioglass^®^ may be used as the GIC glass component [29].

Due to the lack of Al, 45S5 Bioglass^®^ might not have high basicity as other GIC glass compositions [21]. It has been reported that reducing the particle size of the glass component in GICs increases its reactivity [30], resulting in higher setting rates which can make up for the less reactivity caused by the lack of Al in the Bioglass^®^ composition.

The main objective of this study was to develop a remineralizing, aluminum-free GIC consisting of an optimum ratio of 45S5 Bioglass^®^ to its glass-ceramic as the glass component as well as polyacrylic acid aqueous solution. The new resulting GICs exhibited proper mechanical properties comparable to commercially available GICs. We also show that our 45S5 Bioglass^®^-based GIC not only meets the ISO compressive strength requirement but also may improve the remineralizing properties of cements.

## Materials and methods

### Bioglass^®^ synthesis, heat treatment and characterization

The 45S5 Bioglass^®^ used in this research was prepared and donated by Dr. Robert Hill from the Queen Mary University of London. In short, calculated amounts of high-purity precursors were mixed and then melted in a platinum/rhodium crucible at 1380 °C for 1 h using an electric furnace. The resulting melt was then rapidly quenched in deionized water at room temperature. Next, the glass frit was dried and melted again for 30 min. followed by a grinding process using a vibratory mill [19, 29, 31].

To eliminate any internal stress created during the grinding process, the as-received Bioglass^®^ was annealed at 460 °C for 8 h under ambient atmosphere. X-ray diffraction (XRD; PANalytical X'Pert Pro) using Cu Kα radiation (10° < 2θ < 90°) was used to confirm that the amorphous nature of the material was completely retained.

In order to prepare the 45S5 bioglass-ceramic containing combeite, the as-received 45S5 Bioglass^®^ was heat treated through a controlled process [29] to induce 74% crystallinity in its structure which was previously reported to be the optimum crystallinity, leading to the highest mechanical performance when incorporated into GICs [19]. The heat treatment profiles used to obtain various degrees of combeite crystallinity in 45S5 Bioglass^®^ are explained in detail in our previous study [29]. In brief, the heat treatment procedure to obtain 45S5 bioglass-ceramic with 74% crystallinity included a nucleation process at 550 °C for 6 h (dwell time) followed by a growth process at 680 °C for 5 min. (dwell time) [29]. The degree of crystallinity of the resulting heat-treated powder was calculated using its XRD pattern and the intensity of its different characteristic peaks, as explained in detail in reference [29]. The heat-treated glass-ceramic was then lightly ground using an agate mortar and pestle. The particle size distribution of the as-received glass and heat-treated glass-ceramic was measured via laser light scattering particle size distribution analysis (PSA; Horiba LA-920) using isopropyl alcohol as a dispersant and after 5 minutes in an ultrasonic bath. Note that the refractive indices of 1.55 and 1.08 were respectively used for the 45S5 Bioglass^®^ and isopropyl alcohol. Also, the morphology of the as-received glass and heat-treated glass-ceramic particles was examined using a scanning electron microscope (SEM; HITACHI, S-3400N).

### Cement preparation

The glass component of our cements was prepared by mixing of 45S5 Bioglass^®^ and 45S5 bioglass-ceramics (74% crystallinity). In order to investigate the effect of incorporation of bioglass-ceramic, eleven powder mixtures containing 0, 10, 20, 30, 40, 50, 60, 70, 90 and 100 wt% bioglass-ceramic (C0, C10, C20, …, C100; Table 1) were prepared using a Coulter mixer for 30 min. to reach homogeneity. Note that the materials codes and details of the glass powder components are given in Table 1.

**Table 1:** Bioglass^®^ and bioglass-ceramic contents; powder to liquid ratios; and setting times (*n* = 3) of the cements

Cement group	Bioglass-ceramic^a^ (wt%)	Bioglass^®^ (wt%)	P:L (g/g)^b^	Setting time (min.)
C0	0	100	1:0.8	6.5 ± 1.2
C10	10	90	1:0.8	6.8 ± 2.2
C20	20	80	1:0.8	7.9 ± 1.7
C30	30	70	1:0.8	8.7 ± 3.3
C40	40	60	1:0.8	8.6 ± 2.6
C50	50	50	1:0.8	9.1 ± 2.4
C60	60	40	1:0.8	10.6 ± 2.8
C70	70	30	1:0.8	10.8 ± 3.4
C80	80	20	1:0.8	15.4 ± 3.7
C90	90	10	1:0.8	18.1 ± 3.4
C100	100	0	1:0.8	22.7 ± 3.2

^a^As mentioned in previous sections, all bioglass-ceramics used in this study have 74% crystallinity

^b^Note that the liquid component consists of 0.4 g polyacrylic acid and 0.4 g water (50 wt%)

A 50 wt% aqueous solution of PAA, Mw = 240,000 g/mol (Acros Organics) in deionized water was used as the liquid component of the cement. The glass powder components were hand-mixed with the liquid component on a watch glass at different ratios. The powder to liquid ratio (P:L) with the best working and handling properties (P:L = 1:0.8) was found by trial and error among ten tested ratios ranging from 1:0.4 to 1:2.2. Note that all the samples were prepared at room temperature (21 ± 2 °C) throughout this process. Depending on the subsequent characterization technique, different setting procedures were followed as explained in the next section.

### Cement properties

The setting time of each cement was recorded (*n* = 3) according to ISO 9917-1: dentistry-water-based cements [32]. Compression tests were carried out according to the same standard [32]. Cylindrical-shaped cements were made at ambient temperature (21 ± 2 °C) by casting the mixed materials into silicone molds (D = 4 mm and h = 6 mm) followed by an hour incubation at 37 ± 1 °C. Samples were then removed from the molds and incubated in distilled water at 37 ± 1 °C for various durations of 1 h, 1 d, 3 d, 7 d, 14 d, 21 d, and 42 d. After each time point, samples were tested using an Instron 3382 Universal Testing Machine with a 5 kN load cell at a crosshead speed of 0.5 mm/min. The compressive strength (CS) was calculated using the following equation (*n* = 6):

1 $$CS = \frac{{4P}}{{\pi D^2}}$$

where *P* is the maximum applied load (N) and *D* is the diameter of the sample (mm). The fracture surface of the cements was examined by SEM.

A microhardness tester (Mitutoyo MVK-H1) was also used to measure the Vickers microhardness of all the cements by applying a 50 gf load on the surface of each cement for 30 s (*n* = 6).

The resulting cements were characterized by Fourier transform infrared spectroscopy (FTIR, Thermo Scientific, Nicolet iS10) at room temperature over a wavenumber range of 500 to 4000 cm^−1^. Each FTIR experiment was performed at the high resolution of 1 cm^−1^ over 64 scans. However, only the portions of the FTIR spectra containing useful information are presented here. To examine the effect of the powder composition, the FTIR spectra of the C0, C50, and C100 cements after 7 d immersion in distilled water (DW) were obtained and studied. To study the effect of immersion time, the C50 cements, which exhibited the highest mechanical properties among all compositions, were examined by FTIR after 1 d, 7 d, and 21 d immersion in DW. To investigate the early stages of setting, spectra were collected from the C50 composition at 1, 3, 5, 7, 10, 15, 20, and 25 min immediately post mixing (*n* = 3). To monitor the setting progress of the C50 cement, the band intensity ratios between the free carboxyls (COOH) absorption band at ≈ 1650 cm^−1^ to the Ca-polyacrylate (COO^−^ Ca^2+^) absorption bands at ≈1550 cm^−1^ and 1410 cm^−1^ [33–36] were computed from each FTIR spectrum and the differences in the COO^−^ Ca^2+^/COOH band intensity ratios as a function of setting time (1, 3, 5, 7, 10, 15, 20, and 25 min) were plotted.

## Results and discussions

### Bioglass^®^ characterization

**Fig. 1:**
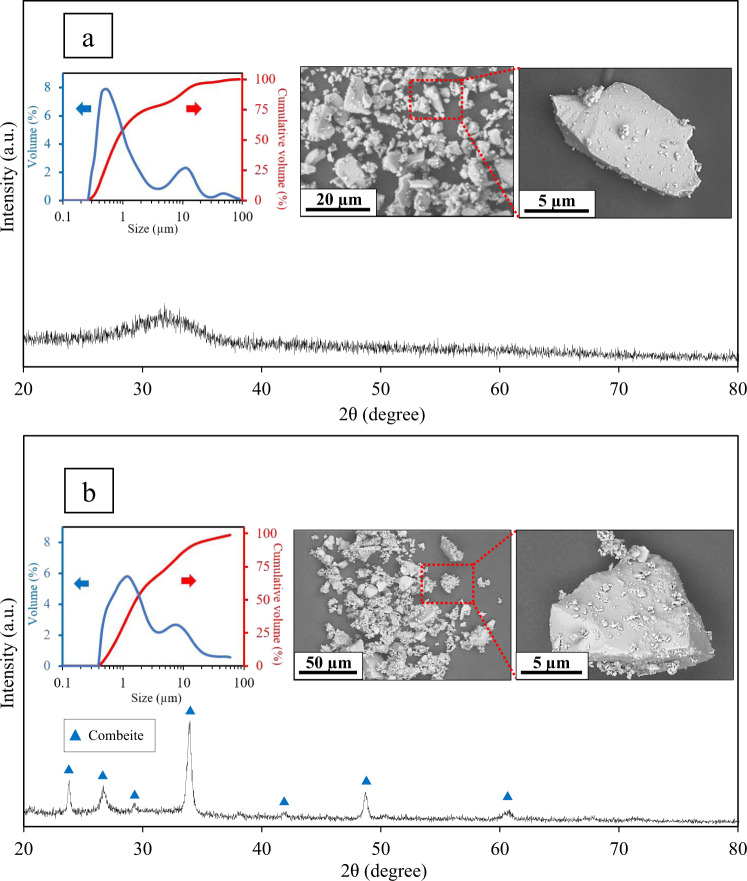
XRD patterns, particle size distributions and SEM micrographs of (**a**) the as-received Bioglass^®^ powder and (**b**) heat treated bioglass-ceramic particles with 74% crystallinity. The XRD patterns are adapted with permission from [29]. Copyright 2018, Elsevier. Note that all these results are obtained from the same batch of bioglass and bioglass-ceramic

The XRD patterns, the PSA results (particle size distribution) and the SEM micrographs of both as-received Bioglass^®^ and the heat treated bioglass-ceramic particles with 74% crystallinity are presented in Fig. 1. The XRD pattern of Fig. 1a confirms that the as-received Bioglass^®^ has retained its amorphous nature after the stress-relief heat treatment at 460 °C as explained in section 2.1. Also, the XRD pattern of Fig. 1b reveals that the bioglass-ceramic powder contains solely combeite crystalline phase. The particle size of Bioglass^®^ powder (Fig. 1a) is in the range of 0.3–90 µm with an average of 4.6 µm. The wide bimodal distribution in this graph consists of two major populations of submicron and micron-sized particles. On the other hand, the particle size distribution of bioglass-ceramic particles (Fig. 1b) shows a slight shift to larger particle size (ranged from 0.3 to 100 µm; mean size = 6.3 µm) while maintaining a similar bimodal distribution. The shift to the larger size and the decrease in intensity of submicron peak can be the result of heat treatment which might lead to slight agglomeration. These characterization results are important since particle size can significantly affect the mechanical properties of cements [30, 37]. GICs prepared by the powders with a bimodal particle size distribution, are shown to have higher fracture toughness and improved workability properties. This is due to the fact that, such distribution leads to a high packing density of the glass particles within the cement matrix in which fine particles mostly provide the reactive surface area required for setting reaction (i.e., faster setting times) whilst coarse particles may mainly contribute to the strengthening mechanisms (i.e., crack deflection) [21, 30, 37, 38]. In the absence of Al as an important element in 3D polysalt formation and hardening of the cements [4], particle size distribution can play a significant role in tailoring the mechanical properties of aluminum-free cements.

In the SEM images (Fig. 1), submicron and micron-sized glass particles can be observed in both as-received Bioglass^®^ (Fig. 1a) and the bioglass-ceramic (Fig. 1b) powders which is consistent with the PSA results. The SEM images also show submicron particles that are agglomerated onto the micron-sized particles which seem to be more in the case of heat-treated powder (Fig. 1b). This may happen due to the higher surface energy of sub-micron particles which increases their tendency to agglomerate [25].

### Cement properties

The powder/liquid (P:L) ratios and setting times of all cements are presented in Table 1. The P:L of 1:0.8 which was the highest ratio that delivered the best working and handling properties, was obtained by trial and error. It has been shown that higher P:L can result in better mechanical performance in GICs due to the development of more polysalt bridges which results in a higher degree of crosslinking [33]. According to this table, the setting time increases with increasing the bioglass-ceramic content of the cements. This can be attributed to the lower reactivity of the bioglass-ceramic particles compared to that of the amorphous Bioglass^®^ [39] resulting in slower reaction with polyacrylic acid (i.e., delayed setting process).

It has been reported that the metallic ions (e.g., Ca^2+^) in 45S5 Bioglass^®^ glass network can be released upon reacting with PAA and take part in setting reactions [21]. The setting times of 45S5 Bioglass**®**-based GICs (Table 1) are longer than the range of 1.5–6 min. required by ISO 9917-1:2002 standard for GICs when used as restorative materials. The prolonged setting times may be the result of the absence of Al component limiting the ability of the cements from forming polysalt bridges at higher rates upon mixing. Even though our GIC still can be considered for other glass ionomer cement applications [32], the resulted setting times can be reduced by manipulating different parameters such as particle size, polyacid composition, etc.

**Fig. 2:**
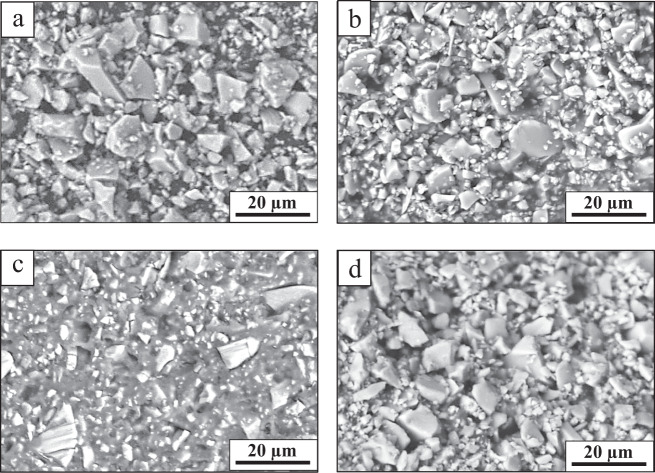
SEM images captured from the surface of (**a**) C0, (**b**) C20, (**c**) C50 and (**d**) C100 cements after setting

Figure 2 shows the SEM micrographs for the C0, C20, C50, and C100 cements after setting. All micrographs show a high packing density in the cements structures which is partly due to the bimodal particle size distribution (Fig. 1). Such distribution of particle size is shown to have a twofold effect on setting time and mechanical properties of the GICs as explained in section 3.1. In the structure of C0 and C20 cements (Fig. 2a, b), more loosely attached particles can be observed. The setting reaction rate for these systems may be too high such that a fraction of the glass particles were remained unreacted (i.e., loosely attached). Interestingly, the SEM images of C100 cement (Fig. 2d) show a similar microstructure containing many loosely attached particles. This is attributed to the fact that the bioglass-ceramic content of C100 cement is less reactive compared to the fully amorphous glass content of other cements [39] resulting in unreacted particles that are poorly attached to the matrix. On the other hand, in the C50 cement micrograph (Fig. 2c), the particles are shown to be more integrated and attached to the cement matrix. Thus, C50 cement containing 50 wt% Bioglass^®^ and 50 wt% bioglass-ceramic with bimodal particle size distributions may present an optimum composition where the submicron Bioglass^®^ particles have sufficient surface area to fully react with PAA, while the larger particles, in particular the less reactive glass-ceramic particles, are allowed to be embedded within the matrix during this setting process. This also explains the high mechanical properties of the C50 cement which is discussed in the next section.

#### Mechanical properties

**Fig. 3:**
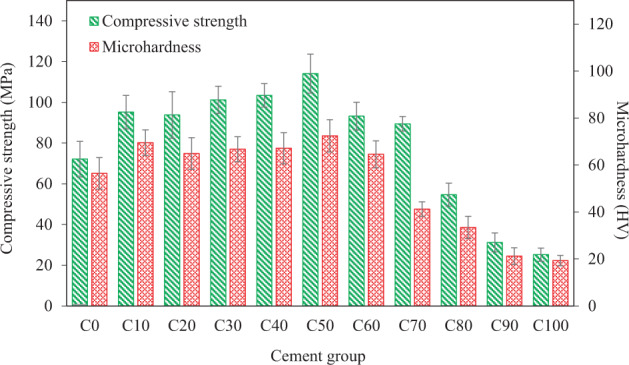
Compressive strength and microhardness of cements containing various amounts of bioglass-ceramic particles (Table 1) after 7 d immersion in distilled water (*n* = 6)

The results of the compression and microhardness tests performed on the cements containing various amounts (Table 1) of bioglass-ceramic particles are presented in Fig. 3. Both compressive strength and microhardness have been improved for all cements containing bioglass-ceramic up to 60 wt% compared to those of C0 cement (i.e. the control cement containing no bioglass-ceramic). Note that the C50 cement exhibits the highest combination of both compressive strength and microhardness among all cement compositions. The strengthening mechanisms that can contribute to the high mechanical properties of this cement are discussed at the end of this section based on SEM images. Higher mechanical properties of the C50 cements can be attributed to the higher strength of the bioglass-ceramic compared to that of the Bioglass^®^ [40] making C50 a cement with a stronger reinforcing agent than those with less bioglass-ceramic content. Besides, C50 was shown to have an optimum composition (50 wt% Bioglass^®^, 50 wt% bioglass-ceramic) leading to a high packing density (Fig. 2c). Cements containing more than 50 wt% bioglass-ceramic in their solid component, exhibit a constant decrease in their compressive strength and microhardness. The reactivity of bioactive glasses is known to decrease upon crystallization [39]. Therefore, bioglass-ceramic is considered less reactive with PAA compared to the Bioglass^®^. Incorporation of the excess amounts of bioglass-ceramic (50 wt% ≤) may interfere with the setting reaction which results in more unreacted particles remaining in the system (e.g., Fig. 2d).

According to ISO 9917-1:2002 standard, the minimum compressive strength for a clinically acceptable GIC as restoratives is 100 MPa [32, 41, 42] which reveals that C30, C40 and C50 cement groups comply with the compressive strength requirements of this standard. A comparison between our GICs and a few commercially available GICs reveals that C50 has higher compressive strength (~114 MPa on the average) than Fuji IX (99 MPa) and Vitro Molar (70 MPa) [43–45]. Also, the microhardness of the C50 cement (~72 HV on the average) is comparable to that of the α-Fil (87 HV), Fuji II (83 HV) and Ketac Molar Easymix (73 HV) [43–45]. Note that all the aforementioned commercial GICs contain Al whereas our GIC is Al-free. Even though the 45S5 Bioglass^®^ composition lacks aluminum and it was not originally designed to be used in GICs, these results show that Al-free 45S5 Bioglass^®^-based cements are promising and can compete with commercially available GICs in terms of mechanical performance.

**Fig. 4:**
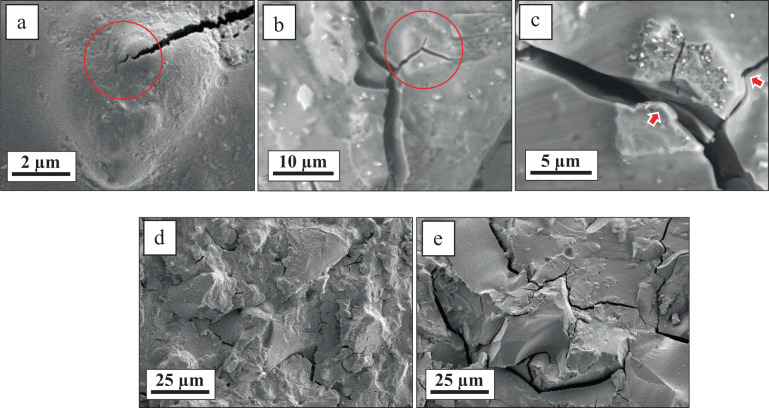
SEM images captured from the fracture surface of the C50 cement after the compression tests (**a**, **b** and **c**). The circles and arrows indicate crack-tip shielding (**a** and **b**) and crack deflection mechanisms (**c**), respectively. Figure 4d, e shows the fracture surface of the C50 cement and a commercial GIC (Chemfil Rock, Dentsply), respectively

To further investigate the active strengthening mechanisms, the fracture surface of the C50 cement, which demonstrated the highest mechanical performance, was analyzed using SEM (Fig. 4). Figure 4a shows a crack that is entering into an embedded submicron particle where it is stopped from further propagation (denoted with a circle) which is known as crack-tip shielding mechanism that can effectively contribute to the improvement of toughness in brittle materials [46]. A similar mechanism can be also observed in Fig. 4b. In this mechanism, the stress at the crack tip is decreased by the formation of a dislocation cloud around the crack tip constraining the crack growth. Figure 4c demonstrates another mechanism known as crack deflection mechanism where a crack deviates from its original path when it is encountered with a reinforcing agent (i.e., a bioglass-ceramic particle). In this mechanism, the increased crack surface due to the deflection, reduces the stress intensity at the crack tip and dissipates its energy [46, 47]. Figure 4d, e shows the fracture surface of the C50 cement and a commercial GIC (Chemfil Rock, Dentsply), respectively. It can be observed that the fracture surface of the C50 cement has a grainy, rough structure similar to that of the commercial GIC revealing the significant fracture surface energy consumed upon failure.

**Fig. 5:**
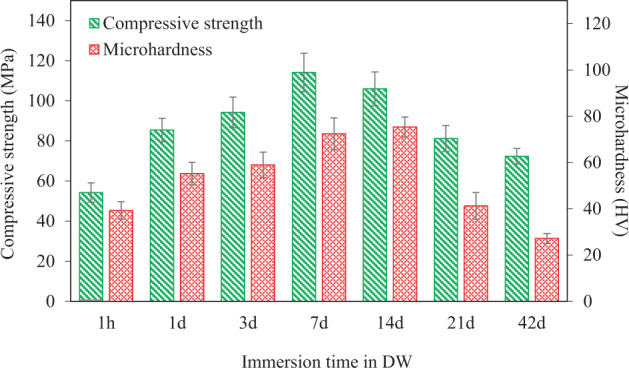
The effect of immersion time in DW on compressive strength and microhardness of the C50 cement (*n* = 6)

In Fig. 5 the effect of immersion time in DW on compressive strength and microhardness of the C50 cement, which exhibited the best mechanical properties (Fig. 3), is presented. Longer immersion in DW has been reported to improve the compressive strength of GICs [48]. During GIC setting, there is a progressive diffusion-controlled process that forms a hard phase due to the continuous crosslinking of the polyacrylate chains. The presence of Si and P within the matrix results in the formation of an inorganic network interpenetrating with the metal (e.g. Ca^2+^) polyacrylate one. Formation of such inorganic network along with the aforementioned hard phase, leads to a gradual improvement in the strength, stiffness, and insolubility of GICs over time, even after the setting [7]. This is consistent with our mechanical properties results (Fig. 5) for the C50 cement showing that immersion in DW up to 7 d increases the compressive strength and microhardness of this cement. However, the compressive strength and microhardness of the C50 cement gradually decrease for immersion times longer than 7 d and 14 d, respectively. This can be attributed to the presence of unreacted particles, which did not fully participate in the setting reaction, within the final cement (previously observed in Fig. 2). Such loosely attached particles may be detached more easily from the matrix over longer immersion times in DW, creating potential crack initiation sites which may result in lower mechanical properties. However, after 42 days immersion in DW, the compressive strength of this cement is still above the standard value of 50 MPa for dental applications such as luting and base/lining [32].

By applying some adjustments, even higher mechanical properties may be obtained for these cements, particularly in longer term. According to the PSA results (Fig. 1a, b), more than 70 vol% of the particles in both 45S5 Bioglass^®^ and bioglass-ceramic particles are submicron. Since these particles provide a large surface area to react with PAA, there may be an insufficient number of ionomer available to effectively hold a large number of submicron particles resulting in those particles remaining unreacted and unattached to the matrix of the cements which lower their final strength. This might be also the reason for the relatively low P:L ratio of the cements in this study (1:0.8) reported in Table 1. It has been previously shown that low P:L ratios, may lead to lower mechanical properties (e.g., decreased compressive strength values) [49]. Having a bimodal particle size distribution consisting of two equally populated submicron and micron-sized ranges, may reduce the number of unreacted particles and allow for higher P:L ratios, all of which potentially can improve the mechanical performance of the cements.

#### FTIR analysis

**Fig. 6:**
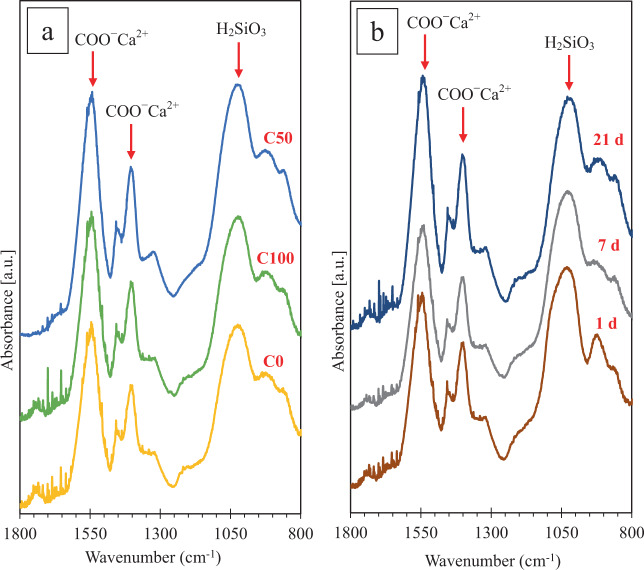
FTIR spectra of the C0, C50, and C100 cements after 7 d immersion in DW (**a**); and the C50 cement after 1, 7, and 21 days of immersion in DW (**b**)

The FTIR spectra showing the effects of powder composition and immersion time on the structure of our Bioglass^®^-based GICs are presented in Fig. 6. The absorption bands at 1410 cm^−1^ and 1550 cm^−1^ respectively correspond to the symmetric and asymmetric stretching vibration of Ca-polyacrylate complex (COO^−^ Ca^2+^) and the peak at 1050 cm^−1^ is assigned to silicic acid (H_2_SiO_3_) formation [33–36]. Ca-polyacrylate formation is the result of PAA ionic crosslinking which utilizes cations (i.e., Ca^2+^) leaching out of the glass particles [50]. Also, silicic acid is the result of the dissolution of SiO_2_ glass network when attacked by PAA [51].

Figure 6a presents the FTIR spectra of the C0, C50, and C100 cements. The spectrum of the C50 cement shows the highest peak intensity for both COO^−^ Ca^2+^ and H_2_SiO_3_, among the three spectra, which may indicate a higher degree of crosslinking that is in line with its superior mechanical properties (Fig. 3). The FTIR spectra of the C50 cement after 1 d, 7 d, and 21 d immersion in DW are presented in Fig. 6b. It can be observed that by increasing the immersion time, the intensity of both COO^−^ Ca^2+^ and H_2_SiO_3_ peaks increases which can be due to the continuous crosslinking of the polyacrylate chains and formation of the hard phase.

**Fig. 7:**
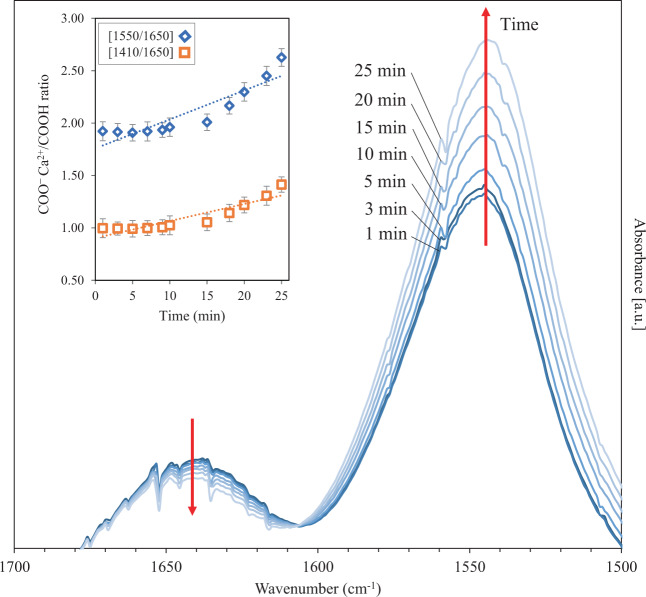
Change in intensity of the absorbance peaks of COO^−^ Ca^2+^ (1550 cm^−1^) and COOH (1650 cm^−1^) during FTIR analysis of the C50 system as a function of time. The subplots in the upper left of spectra depict the ratio of 1550 cm^−1^ to 1650 cm^−1^ () and 1410 cm^−1^ to 1650 cm^−1^ () band intensities (*n* = 3). Note that the peak assigned to the symmetric vibration band of COO^−^ Ca^2+^ (1410 cm^−1^) which was used in the subplot is not presented here

Using FTIR, the complex formation process of our GICs is assessed in situ over the initial 25 minutes of setting (Fig. 7) through monitoring the changes in absorbance ratio of complexed to free carboxyls [34–36]. Figure 7 shows the change in the intensity of the COOH (1650 cm^−1^) and the COO^−^ Ca^2+^ (1550 cm^−1^) peaks for our Bioglass^®^-based GIC (C50) over time. It appears that, the intensity of the COO^−^ Ca^2+^ peak increases overtime at the expense of the COOH peak. This gradual time-dependent change can be an indication of the progression of the acid-base reaction until the GIC sets [33–36]. The released Ca^2+^ ions from the glass particles start to create salt bridges which crosslink the polyacrylate chains and lead to a decrease in COOH (1650 cm^−1^) peak intensity. Simultaneously, the intensity of peaks corresponding to the symmetric (1410 cm^−1^) and asymmetric (1550 cm^−1^) Ca-polyacrylate (COO^−^ Ca^2+^) bonds increases. The progression of the GIC setting reaction is further assessed by plotting the COO^−^ Ca^2+^/COOH ratios (i.e., the ratio of 1550 cm^−1^ to 1650 cm^−1^ and 1410 cm^−1^ to 1650 cm^−1^) over time (the subplot in Fig. 7). The COO^−^ Ca^2+^/COOH ratio increases over time indicating the increase in concentration of the crosslinked carboxylate groups and the progressive nature of the setting reaction in our GICs [34, 35].

## Conclusions

In this study, we developed an Al-free 45S5 Bioglass^®^-based GIC with standard compressive strength comparable to that of the commercially available GICs. We showed that the cements with the solid component containing 50 wt% Bioglass^®^ and 50 wt% bioglass-ceramic (74% crystallinity) exhibited the highest combination of compressive strength and microhardness. Strengthening mechanisms such as crack deflection and crack-tip shielding were found to be activated enhancing the mechanical properties of this group of GICs. Also, cements with lower (e.g., 10 wt%) and higher (e.g., 100 wt%) bioglass-ceramic contents showed many loosely attached particles in their microstructure acting as crack initiation sites. It was also discussed that the bimodal particle size distribution of the solid component in these GICs may have contributed to their high packing density and structural integrity after setting where smaller particles mostly take part in the setting reaction while larger particles participate in strengthening mechanisms e.g., crack deflection. Supplementary in vitro and in vivo tests are required in the future to further study these 45S5 Bioglass^®^-based GICs and their long-term performance as dental restorative and/or bone cements.
